# Mountain pine beetle host-range expansion threatens the boreal forest

**DOI:** 10.1111/j.1365-294X.2011.05086.x

**Published:** 2011-05

**Authors:** Catherine I Cullingham, Janice E K Cooke, Sophie Dang, Corey S Davis, Barry J Cooke, David W Coltman

**Affiliations:** * Department of Biological Sciences, CW405 Biological Sciences Building, University of AlbertaEdmonton, AB T6G 2E9, Canada; † Northern Forestry CentreCanadian Forest Service, 5320-122nd Street, Edmonton, AB T6H 3S5, Canada

**Keywords:** host-range expansion, hybrid, jack pine, lodgepole pine, mountain pine beetle

## Abstract

The current epidemic of the mountain pine beetle (MPB), an indigenous pest of western North American pine, has resulted in significant losses of lodgepole pine. The leading edge has reached Alberta where forest composition shifts from lodgepole to jack pine through a hybrid zone. The susceptibility of jack pine to MPB is a major concern, but there has been no evidence of host-range expansion, in part due to the difficulty in distinguishing the parentals and their hybrids. We tested the utility of a panel of microsatellite loci optimized for both species to classify lodgepole pine, jack pine and their hybrids using simulated data. We were able to accurately classify simulated individuals, and hence applied these markers to identify the ancestry of attacked trees. Here we show for the first time successful MPB attack in natural jack pine stands at the leading edge of the epidemic. This once unsuitable habitat is now a novel environment for MPB to exploit, a potential risk which could be exacerbated by further climate change. The consequences of host-range expansion for the vast boreal ecosystem could be significant.

## Introduction

Increasing global temperatures have lead to elevational and/or latitudinal shifts in species ranges (Parmesan 1996; Bale *et al.* 2002; Walther *et al.* 2002). This is especially true for insects because they are poikilothermic organisms and therefore quickly respond to changes in their thermal environment. For forest insect pests this is expected to result in negative economic and ecological impacts (Ayres & Lombardero 2000; Logan *et al.* 2003; Battisti *et al.* 2006). Range shifts can expose novel habitats, where naïve hosts may not have evolved appropriate defences to ward off attack (Cudmore *et al.* 2010). Changes in climate suitability may go beyond the range of the host species, which could mean a dead end for the insect. However hybrid zones between forest species can provide a phenotypic stepping-stone which can help mediate range expansion into a new host (Floate & Whitham 1993; Pilson 1999).

The mountain pine beetle (MPB; *Dendroctonus ponderosae* Hopkins) is a bark beetle indigenous to western North America that primarily feeds on lodgepole pine (*Pinus contorta* Dougl. ex Loud. var. *latifolia*), but also feeds on sugar pine (*P. lambertiana* Dougl.), western white pine (*P. monticola* Dougl. Ed. D. Don) and ponderosa pine (*P. ponderosa*, P. Laws. Ex C. Laws; Safranyik & Carroll 2006). Beetle populations typically infest damaged trees or trees with compromised defence capacity; however, given the right conditions they can erupt into large-scale outbreaks and cause significant losses of mature healthy stands (Safranyik & Carroll 2006). The most recent outbreak has affected over 14 million hectares of forest land in western Canada (Nealis & Peter 2008) with considerable losses recorded in the USA (Hicke *et al.* 2006). This is the largest outbreak that has been documented since record taking began approximately 125 years ago (Taylor & Carroll 2004; Raffa *et al.* 2008) and brings negative economic impacts, inefficient nutrient cycling and carbon sequestration, and reduced biodiversity (Ayres & Lombardero 2000; Kurz *et al.* 2008).

Until recently, the range of MPB in Canada has been primarily restricted to the British Columbia interior due to physiological restrictions for MPB (Bentz *et al.* 2010). Colder temperatures at higher elevations and increasing latitudes can affect their synchrony and over-winter survival, suppressing population growth at their range limit (Logan & Powell 2001). However, a suite of studies examining future climate change scenarios have predicted a geographic range shift into previously marginal habitat (Logan & Bentz 1999; Logan & Powell 2001; Carroll *et al.* 2003; Fauria & Johnson 2009). As predicted, the beetle has traversed the Rocky Mountains and has reached the eastern edge of the range of lodgepole pine in north-central Alberta (Robertson *et al.* 2009; Bentz *et al.* 2010) where forest composition shifts to jack pine (*P. banksiana*, Lamb) through a hybrid zone (Zavarin *et al.* 1969; Pollack & Dancik 1985). This hybrid zone could conceivably help mediate MPB host-range expansion into jack pine. Jack pine is a boreal species whose range extends from Alberta to Nova Scotia. There has been no record of MPB infection in natural hybrid or jack pine stands (Bentz *et al.* 2010; Safranyik *et al.* 2010). However, there is considerable evidence to suggest hybrids and jack pine would be suitable hosts for MPB reproduction (reviewed in Safranyik *et al.* 2010). Given the close evolutionary relationship between lodgepole and jack pine (Wheeler *et al.* 1983), together with the ability of MPB to attack more distantly related pine (Bentz *et al.* 2010) and instances of MPB attack on jack pine in nursery settings (Furniss & Schenk 1969; Safranyik *et al.* 2010), hybrids and jack pine are likely to be compatible hosts for MPB. Consequently, the arrival of MPB in north-central Alberta has raised major concerns regarding the potential for further expansion to the vast boreal forest.

**Fig. 1:**
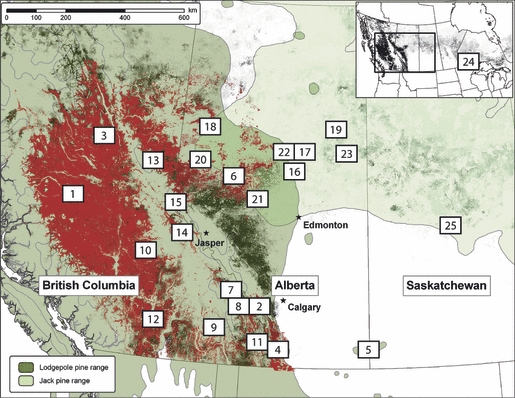
Sampling locations for lodgepole pine, jack pine and hybrids across western Canada analysed at 12 microsatellite loci. MPB attack data from 1958 to 2009 for British Columbia and from 1975 to 2009 for Alberta are indicated in red (Thandi & Taylor unpublished data), and approximate pine volume is shaded in green, where maximum densities are over 500 m^3^/ha (Yemshanov & McKenney unpublished data). Top-right inset of North America illustrates the location of the Ontario/Minnesota samples. Range distributions for jack pine and lodgepole pine were obtained from USGS (http://esp.cr.usgs.gov/data/atlas/little/, accessed 29 July 2010) and are based on Little (1971).

Evidence for host-range expansion by MPB to jack pine and hybrids has been difficult since it has not been possible to reliably distinguish lodgepole and jack pine from their hybrids (Zavarin *et al.* 1969; Pollack & Dancik 1985; Rweyongeza *et al.* 2007). Seed and cone morphometry are the current criteria for distinguishing species (Wheeler & Guries 1987); however, considerable morphological variation in the hybrid zone makes positive identification difficult (Rweyongeza *et al.* 2007). Previous efforts to identify hybrids using molecular approaches including chemical profiles, allozymes, organellar DNA, random amplified polymorphism and restriction fragment length polymorphisms (Pollack & Dancik 1985; Wheeler & Guries 1987; Dong & Wagner 1993; Yang *et al.* 2007) have also been ineffective in resolving hybrids from parents in the hybrid zone. Microsatellites have been effectively utilized for a variety of taxa where hybrids have been difficult to distinguish from parentals using other characters (Thulin *et al.* 2006; Burgarella *et al.* 2009; Quintela *et al.* 2010). For that reason, our first objective was to develop a panel of microsatellite markers that amplify in both species and test their efficacy to distinguish jack pine, lodgepole pine and their hybrids. We verified our ability to accomplish this by analysing simulated datasets containing multiple levels of admixture generated using pure lodgepole pine (central and southern British Columbia) and jack pine (Saskatchewan and Ontario) as benchmarks. Due to the level of differentiation described between these species (*F*_ST_ = 0.108, Wheeler & Guries 1987; *G*_ST_ = 0.247, Ye *et al.* 2002) we expected accurate resolution of first and second generation hybrids, however, we anticipated diminishing power with advanced generations of backcrossing given these species are closely related (Wheeler *et al.* 1983). Our second objective was to analyse samples collected from MPB attacked and un-attacked trees from the region of MPB expansion including the leading edge in north-central Alberta where the ranges of lodgepole pine and jack pine overlap (Fig. 1). We classified the hybrid and species status of the trees, focusing on MPB-attacked individuals sampled at the leading edge of the expansion based on the information from our simulations and species assignment analysis. We hypothesized that some of the attacked trees in these regions would be jack pine because of the geographic location and the reported susceptibility of jack pine to MPB in nursery settings (Furniss & Schenk 1969; Safranyik *et al.* 2010).

## Methods

### Sample collection

Foliage samples were collected from lodgepole pine within 10 locations in British Columbia (*n*= 160), from 14 jack pine locations in Ontario and Minnesota (*n* = 70) and one location in Saskatchewan (*n* = 43), and from lodgepole pine, jack pine and putative hybrids within 12 locations in Alberta (*n* = 381) (Table 1, Fig. 1). In British Columbia and Alberta both MPB attacked and non-attacked trees were sampled. The majority of the foliage samples were collected during two time periods, from February 2007 to May 2007 and September 2007 to April 2008. Stands for sampling were selected on the basis of aerial and ground survey data of MPB attack provided by Alberta Sustainable Resource Development (ASRD). Aerial surveys were conducted in the late summer and early fall each year by ASRD to identify newly attacked trees by the presence of fading foliage, an indicator of MPB infestation. The majority of aerially identified trees were subsequently ground-truthed by ASRD. For each sampling period, waypoints from the most recently available aerial survey were used to locate candidate trees for sampling. Based on 2009 survey data, additional foliar samples from putative jack pine trees were collected in April and May 2010 in the easternmost extent of detected MPB attack by ASRD (*n* = 6, ‘Smith’–Table 1, Fig. 1) and by the Canadian Forest Service (*n* = 18, ‘Wildwood’–Table 1, Fig. 1).

**Table 1:** Sample size (*N*), number of effective alleles (Ne), observed heterozygosity (H_O_), expected heterozygosity (H_E_) and fixation index (F) calculated for lodgepole pine (Pl), jack pine (Pj) and their hybrids at each location across our study area, the numbers (‘Pop’) correspond to the locations in Fig. 1. Expected species composition is included based on Little's (1971) distribution maps. All measures were calculated in GenAlEx 6.4 (Peakall & Smouse) and were estimated without locus Pcon54

Pop	Location	Prov.	Species	N	Na	Ne	Ho	H_E_	F
1	Bulkley	BC	Pl	3	3.63	3.23	0.667	0.770	−0.072
2	Canmore	AB	Pl	28	12.45	7.24	0.765	0.838	0.066
3	Plateau	BC	Pl	4	4.36	3.50	0.705	0.786	−0.046
4	Crowsnest Pass	AB	Pl	12	8.36	5.73	0.744	0.826	0.054
5	Cypress Hills	AB/SK	Pl	12	7.72	5.27	0.757	0.805	0.000
6	Fox Creek	AB	Pl	16	10.00	5.98	0.746	0.809	0.040
7	Golden	BC	Pl	38	13.18	7.69	0.725	0.828	0.109
8	Kootenay/Yoho	BC	Pl	20	11.36	6.30	0.720	0.817	0.098
9	Nelson	BC	Pl	3	4.18	3.64	0.727	0.836	−0.061
10	Prince George	BC	Pl	5	5.36	3.96	0.595	0.791	0.178
11	Sparwood	BC	Pl	29	12.90	6.81	0.711	0.817	0.112
12	Okanagan	BC	Pl	7	5.45	4.24	0.672	0.787	0.081
13	Tumbler Ridge	BC	Pl	28	12.27	6.69	0.704	0.821	0.123
14	Valemount	BC	Pl	23	11.36	6.70	0.671	0.815	0.156
15	Willmore-Kakwa	AB	Pl	21	11.18	6.63	0.711	0.834	0.110
16	Smith	AB	Pj × Pl	6	4.54	3.28	0.539	0.655	0.067
17	Wildwood	AB	Pj × Pl	18	10.81	6.89	0.764	0.855	0.076
18	Fairview	AB	Pj × Pl	27	12.18	7.61	0.753	0.847	0.091
19	FtMcMurray	AB	Pj × Pl	89	12.54	4.12	0.571	0.659	0.131
20	Grande Prairie	AB	Pj × Pl	30	13.09	7.49	0.707	0.847	0.150
21	Hinton	AB	Pj × Pl	8	6.90	5.01	0.727	0.822	0.048
22	Wabasca	AB	Pj × Pl	34	13.63	8.36	0.761	0.856	0.097
23	Conklin	AB	Pj	104	12.00	3.83	0.548	0.610	0.113
24	Ontario/Minnesota	ON/MN	Pj	70	7.45	3.56	0.536	0.596	0.103
25	Saskatchewan	SK	Pj	43	8.36	3.71	0.537	0.614	0.096
	Average				9.415	5.503	0.683	0.782	0.077

Prior to sampling, each tree was first confirmed for MPB attack and colonization by identification of diagnostic entrance holes in the bark, followed by bark removal around the area of these entrance holes to confirm the presence of MPB larval galleries, as opposed to galleries created by other species such as *Ips pini* Say, which co-occur in this region. For trees sampled in 2010, the existence of pupal chambers was specifically noted as proof that the host is suitable for completing development of all feeding larval stages. Further development of these pupae had likely not occurred because of the time of year that the samples had been taken. In all cases, foliage was collected from the crown using pole pruners or a shotgun. For samples collected in 2007 and 2008, MPB (typically as larvae) from attacked trees were sampled simultaneously for genetic analysis (Samerasekera *et al.*, unpublished). All sampled trees were geo-referenced using Garmin GPS units (Garmin International, Olathe, KS, USA). Samples were stored in coolers until they could be brought to the laboratory, where they were processed and stored at –20 °C or –80 °C until DNA extraction could be performed.

### DNA extraction and genotyping

Genomic DNA was isolated from ground needle tissue using a CTAB (hexadecyl trimethyl ammonium bromide) protocol modified from Chang *et al.* (1993), according to Roe *et al.* (2010). Additional changes include 5 μL of RNase A (70 units/mg protein, Sigma) being added to each sample along with the CTAB buffer. As well, incubation time at 65 °C was increased to 2 h and all centrifugation steps were performed at 5800 ***g*** with the duration of the final two centrifugation steps being increased to 5 min. Pellets were resuspended in 125 μL Milli-Q water.

We screened twenty-three microsatellite loci that were previously tested in lodgepole pine (Auckland *et al.* 2002) and found 10 to be polymorphic in a panel of eight individuals of each species (Appendix I). Additional loci were isolated from a microsatellite enriched (GT_*n*_/CT_*n*_) library constructed from a single lodgepole pine individual using the methods of Glenn & Schable (2005). We selected 368 clones and obtained bi-directional sequences using T3 and T7 primers using BigDye v3.1 sequencing chemistry (Applied Biosystems, Carlsbad, CA, USA) and resolved on a 3730 DNA Analyzer (Applied Biosystems). Sequences were aligned in SeqMan (LaserGene; DNASTAR, Madison, WI, USA) resulting in 222 contigs. Twenty-five contigs were selected that contained long uninterrupted (11–33) repeats with sufficient flanking sequence for primer design. Amplification primers were designed using Primer3 with the default parameters except the optimal *T*_m_ was set to 56 °C and the maximum *T*_m_ difference between pairs of primers was restricted to 1 °C (Rozen & Skaletsky 2000). Two loci were retained (GENBANK accession numbers: HQ404301– 2) that were polymorphic in both species and had clean amplification products (Appendix I).

Genotyping was completed for all individuals at 12 microsatellite loci. These loci were amplified in two single, and five multiplex 15 μL reactions (A–G; Appendix I) containing: ∼400 ng DNA (∼200 ng DNA for the single reactions), 1X PCR Buffer, 160 μM each dNTP, 1% dimethyl sulfoxide (volume to volume), 1U Taq DNA polymerase (AB), and optimized MgCl_2_ and primer amounts (Appendix I). Amplifications were completed using an Eppendorf Mastercycler using the following cycling parameters: 94 °C for 5 min, 33 cycles of 94 °C for 30 s, 56 °C for 30 s, and 72 °C for 15 s, and a final extension at 72 °C for 30 min. These reactions were co-loaded into three injections (1–3; Appendix I) on an ABI 3730 DNA Analyzer and genotyped using GeneMapper software (Applied Biosystems) with allele sizes being determined relative to GeneScan-500LIZ (Applied Biosystems). Genotyping error rate was quantified by running duplicate genotypes for 46 samples.

### Diversity measures

We assessed our microsatellite scoring for stutter errors, large-allele drop-out and null alleles using microchecker (Oosterhout *et al.* 2004); any loci with scoring issues were removed from further analyses. Hardy–Weinberg equilibrium and linkage disequilibrium was assessed in genepop 4.0 (Raymond & Rousset 1995; web version, http://genepop.curtin.edu.au/) within locations (where *n* ≥ 20) for each species (hybrids excluded) as defined by analysis of outputs from Newhybrids and Structure (see Results section). Significance was assessed using Bonferroni corrected alpha values for multiple comparisons (*α* = 0.05, Rice 1989). Standard measures of allelic diversity including number of alleles, effective number of alleles (defined as the number of alleles with equal frequency that would achieve the observed level of diversity, Hedrick 2000) and observed and expected heterozygosities (H_O_ and H_E_, respectively) were calculated in GenAlEx 6.0 (Peakall & Smouse 2006). Allelic richness and private allelic richness corrected for sample size differences were calculated in hp-rare 1.0 (Kalinowski 2005). All measures were estimated for the entire data-set, for each species (hybrids excluded) once identified, and basic diversity measures were calculated for each location. In addition, we calculated the level of differentiation (*F*_ST_) at several hierarchical levels: among locations in the entire dataset, between species and among locations within species, using GenAlEx.

### Species identification

**Fig. 3:**
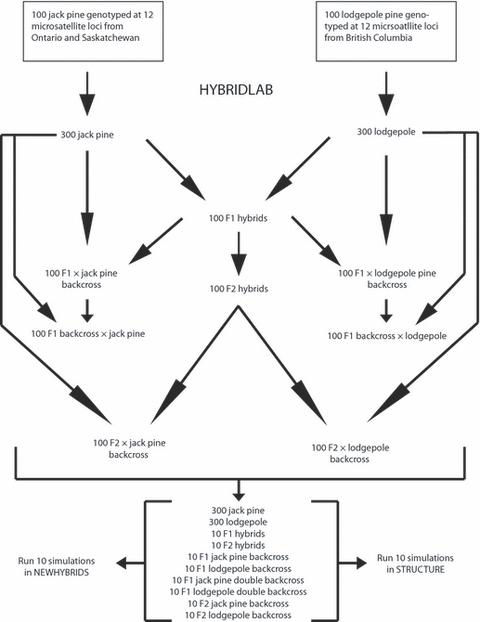
Work-flow for generating genotypes for simulations to assess the capability of 12 microsatellite loci to resolve the species identify of jack pine, lodgepole pine and their hybrids.

The detection of hybrid classes will depend on the degree of differentiation between the parental species and the loci used (Anderson & Thompson 2002; Vähä & Primmer 2006). Previous studies that have assessed hybrid zones and the resolving capacity of microsatellites have included only first (F1) and second generation hybrids (F2, F1 backcrosses) in their simulations (Thulin *et al.* 2006; Vähä & Primmer 2006; Burgarella *et al.* 2009; Quintela *et al.* 2010). We also included a third generation of hybrids (F2 backcrosses and F1 double backcrosses) to assess our ability to resolve advanced introgression. We simulated five datasets using Hybridlab ver. 1.0 (Nielsen *et al.* 2006). Hybridlab was developed to create artificial parental and hybrid genotypes to evaluate the power to correctly identify hybrids. As input, we used genetic profiles from 100 jack pine from Ontario and Saskatchewan and 100 lodgepole pine from British Columbia (we selected samples far removed from the hybrid zone) to represent the microsatellite allele frequency variation for each species. For each dataset we simulated profiles for 300 jack pine and 300 lodgepole pine; we chose 300 as we felt this would simulate our dataset closely. Using these 600 simulated genotypes we generated 100 F1 hybrids. With these hybrid profiles we were able to simulate F2 hybrids, F1 × jack pine (F1Pj), F1 × lodgepole pine (F1Pl), F2 × jack pine (F2Pj), F2 × lodgepole pine (F2Pl), F1-jackpine × jack pine (F1Pj-Pj), and F1-lodgepole pine × lodgepole pine (F1Pl-Pl) (Fig. 3). Each dataset for hybrid analysis was comprised of simulated genotypes for 300 jack pine and 300 lodgepole pine and 10 individuals from each hybrid class: F1, F2, F1Pj, F1Pl, F2Pj, F2Pl, F1Pj-Pj and F1Pl-Pl. We then analysed these datasets with two different Bayesian methods (Structure and Newhybrids) to establish a threshold (*Q*_*T*_) for assigning parental and hybrid status. We used both Bayesian methods in our approach to maximize the accuracy of assigning individuals (Thulin *et al.* 2006; Vähä & Primmer 2006; Burgarella *et al.* 2009).

In Newhybrids v. 1.0 (Anderson & Thompson 2002), we implemented the model with uninformative priors for both the allele frequency and admixture distributions. For each individual Newhybrids calculates the probability of belonging to the parental categories and each specific hybrid classes (e.g. F1, F2, F1 backcross). However, assignment to a specific hybrid class may be uncertain in which case the probability is often split among the hybrid classes (Burgarella *et al.* 2009). Therefore, to assign hybrids we used two different approaches; if the assignment to a hybrid class was of high probability (i.e. an assignment to the F1 hybrid class with over 0.9 probability) we took the assignment. However, if we found the probability was divided among the hybrid classes, we summed the probability over classes, and if the sum was ≥ 0.9 we considered this a hybrid with class undefined. To assign a *Q*_T_ for the parental categories we looked at the range and average assignment across all individuals. We ran each data-set through ten simulations with a burn-in of 50 000 and 500 000 Markov-chain Monte Carlo sweeps for data collection and we did not use prior population information as species designations were not included for the collected samples.

In Structure 2.3.1 (Pritchard *et al.* 2000; Falush *et al.* 2003, 2007), we set *K* = 2, and ran the admixture model with uncorrelated allele frequencies, inferring lambda for each population 10 times. The algorithm was run for 550 000 Markov-chain Monte Carlo sweeps with a burn-in of 50 000 and 500 000 for data collection. We used Clumpp (Jakobsson & Rosenberg 2007) to summarize the ten iterations for each of the five simulated data-sets then looked at the *Q* values for each cluster to determine the most appropriate *Q*_T_. Again, we looked at the range and average of *Q* values for each class as we did with Newhybrids. We assessed the standard deviation among individuals across the 10 iterations for each of the five simulated datasets to determine the margin of error for both programs. We also compared the outputs from both programs for each simulated dataset to assess the level of agreement between the methods.

We analysed our 678 genotyped samples in Newhybrids and Structure using the same parameters and summary methods as our simulations. Our choice of the most appropriate *Q*_T_ for assigning species class was based on the simulations (see Results section). The results from the two programs were compared and a final species class was assigned based on a combination of the two. If the assignment between the two programs did not agree we made a decision based on the *Q* values from the simulations (see Results section).

## Results

### Sample collection

**Fig. 2:**
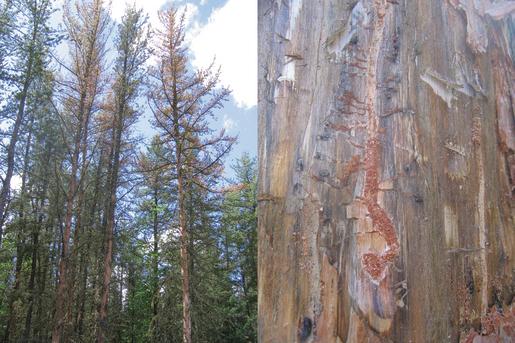
Images from putative jack pine attacked by MPB in north-central Alberta. Left panel shows the red needles indicating tree die-off typical of trees one-year after MPB infection. Right panel shows a set of well-developed larval galleries, including a pupal chamber, indicating successful completion of larval development. Adult beetles from the parental generation (bottom left) occupy a different gallery. Images courtesy of James D. Weber, Canadian Forest Services.

Six-hundred and seventy-eight individuals were used for genotyping (Dryad entry doi:10.5061/dryad.8677), 154 of these represented individuals that had been successfully attacked by MPB. In the putative jack pine sampled in 2010, signs of successful reproduction were observed where pupal chambers were present (Fig. 2), which indicates the eggs hatched and the insects completed all larval stages.

### Diversity measures

Individuals were genotyped at 12 microsatellite loci with less than 2% missing data (555 complete profiles, 101 missing one locus and 22 missing two loci). Our genotyping error rate was very low at 0.8%. Some loci were not in Hardy–Weinberg equilibrium at the location level (Appendix II) with a homozygote excess, however only the locus Pcon54 was consistently and extremely out of Hardy–Weinberg equilibrium, therefore we removed this locus from analyses. After Bonferroni correction we did not find any pairs of loci in linkage disequilibrium in lodgepole pine, and only two comparisons for jack pine. Genetic diversity was high among locations, average H_O_ = 0.683 (Table 1). Genetic diversity measured for each species across loci by H_O_, H_E_, allelic richness and private allelic richness was higher in lodgepole pine (Appendix III). Across the entire dataset, differentiation among locations (*F*_ST_ = 0.125) and between species (*F*_ST_ = 0.133) was high. Within species however, differentiation among locations was very low (*F*_STlodgepole_ = 0.033, *F*_STjack_ = 0.016). However, there were some significant pair-wise comparisons; most notably for jack pine, where all locations were differentiated from Ontario/Minnesota.

### Species identification

**Fig. 4:**
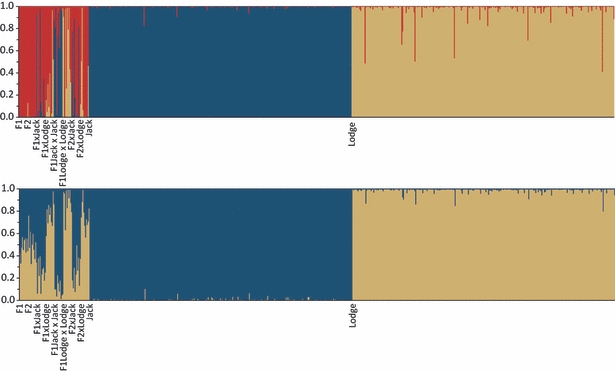
Ancestry plots from simulated lodgepole pine (tan), jack pine (blue), and various hybrid crosses (red for Newhybrids) generated in Newhybrids (top) and Structure (bottom).

Across the five simulated datasets the assignment of individuals to their correct class was never less than 96% using either assignment method (Fig. 4, Table 2). The calculated proportion of ancestry for both programs were highly consistent, the standard deviation among individuals across the ten iterations for each simulated dataset were extremely low (Structure = 0.00014, Newhybrids = 0.00003). As well, the consistency in assignment across the programs was also very high with few discrepancies (Table 3). For both methods detection of 1st generation hybrids was 100%, for 2nd generation hybrids this decreased to 87% and 81% and for 3rd generation hybrids this decreased further to 68% and 63%, in Newhybrids and Structure, respectively (Table 2). Based on the results from Newhybrids the most accurate method to assign hybrids was to sum the estimated proportions across the hybrid categories. When we used a *Q*_T_ of 0.9 for the parental and hybrid classes in newhybrids, similar to other studies (Thulin *et al.* 2006; Vähä & Primmer 2006; Burgarella *et al.* 2009; Quintela *et al.* 2010), we found a large number of individuals that did not assign to any category. However, over 98% of these individuals with *Q*_T_ < 0.90 were hybrids, therefore we used a *Q*_T_ ≥ 0.9 to assign pure species, and all other individuals were assigned hybrid status.

**Table 2:** Accuracy of assignment among five simulated datasets of lodgepole pine, jack pine and their hybrids using Newhybrids 1.0 (NH, Anderson & Thompson 2002) and Structure 2.3.1 (STR, Pritchard *et al.* 2000; Falush *et al.* 2003, 2007). Hybrid categories are as follows: 1st Gen – F1, 2nd Gen – F2 and F1 backcrosses, and 3rd Gen – F2 backcrosses and F1 double backcrosses

	Sim 1		Sim 2		Sim 3		Sim 4		Sim 5		Average	
						
Class	NH	STR	NH	STR	NH	STR	NH	STR	NH	STR	NH	STR
1st Gen	1.00	1.00	1.00	1.00	1.00	1.00	1.00	1.00	1.00	1.00	1.00	1.00
2nd Gen	0.90	0.90	0.90	0.87	0.83	0.63	0.80	0.79	0.93	0.90	0.87	0.81
3rd Gen	0.73	0.65	0.63	0.60	0.73	0.70	0.65	0.55	0.65	0.63	0.68	0.63
Hybrid Avg	0.83	0.79	0.78	0.75	0.80	0.71	0.75	0.69	0.80	0.78	0.79	0.74
Jack	1.00	1.00	1.00	1.00	1.00	1.00	0.99	1.00	1.00	1.00	1.00	1.00
Lodgepole	0.96	0.99	0.98	1.00	0.98	0.99	0.97	0.98	0.96	0.99	0.97	0.99

**Table 3:** Classification of simulated data sets (Sim01–Sim05) and trees sampled in Alberta, British Columbia, Ontario and Saskatchewan (Data) based on outputs from Newhybrids (NH) and Structure (ST) to jack pine, lodgepole pine and hybrids. There was a high level of agreement between the two methods (8–16 discrepancies)

	Sim01			Sim02			Sim03			Sim04			Sim05			Data		
						
NHST	Jack	Lodge	Hybrid	Jack	Lodge	Hybrid	Jack	Lodge	Hybrid	Jack	Lodge	Hybrid	Jack	Lodge	Hybrid	Jack	Lodge	Hybrid
Jack	306	0	4	302	0	6	310	0	0	303	0	2	307	0	4	294	0	1
Lodge	0	295	9	0	304	6	0	301	8	0	307	8	0	298	12	0	287	2
Hybrid	0	0	66	0	0	62	0	0	61	0	0	60	0	0	59	6	1	87

Using Structure, we found that *Q*_T_ ≥ 0.9 gave the most accurate pure assignment for simulated data, but cases of advanced introgression were difficult to detect (Fig. 4). The average disagreement between Structure and Newhybrids across the five simulations was 1.4%. All of these discrepancies were either hybrids correctly assigned by Newhybrids, or pure individuals correctly assigned by Structure. Based on these discrepancies we developed a set of rules for assigning individuals when the methods had conflicting results: (i) if the probability of being a hybrid in Newhybrids is ≥ 0.9 and the probability of being a parental in Structure is > 0.9 but < 0.95 the individual was assigned hybrid status. (ii) If the probability is most likely to be a pure parental in Newhybrids but < 0.9 and in Structure is > 0.95, then the individual is assigned to the parental category.

**Fig. 5:**
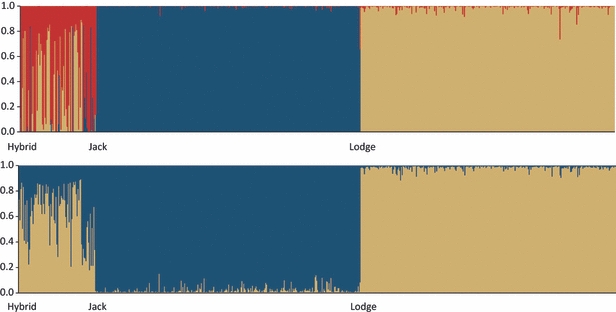
Ancestry plots generated in Newhybrids (top) and Structure (bottom) for 678 trees sampled across Alberta, British Columbia, Ontario, Minnesota, and Saskatchewan illustrating lodgepole pine (tan), jack pine (blue) and hybrid (red for Newhybrids) ancestry.

**Fig. 6:**
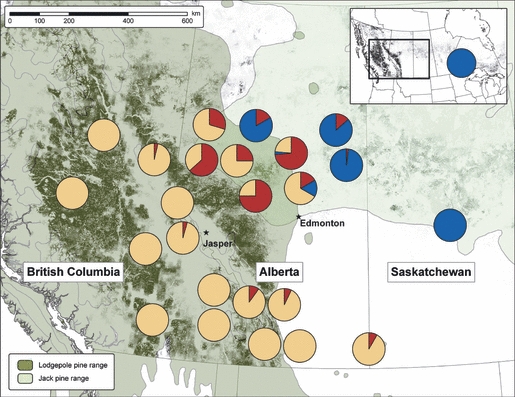
Proportion of lodgepole pine (tan), jack pine (blue), and hybrids (red) at 24 locations in western Canada. Assignment to species categories was based on results from Newhybrids and Structure. Range distributions for jack pine and lodgepole pine were obtained from USGS (http://esp.cr.usgs.gov/data/atlas/little/, accessed 29 July 2010) and are based on Little (1971).

We were able to clearly delineate the two species and our power to detect hybrid individuals was 0.74 averaged across three generations of hybrids in the simulated data (Table 2). We therefore used a *Q*_T_ ≥ 0.9 in both Structure and Newhybrids for the assignment of pure lodgepole pine and jack pine, and considered the unassigned individuals as hybrids. The two assignment methods agreed for 668 of the 678 samples analysed (Table 3). The 10 disagreements were resolved using the rules that we developed based on the simulations (see above). The final breakdown of assignment for our sample data was 87 hybrids, 301 jack pine and 290 lodgepole pine (Figs 5 and 6). Ancestry of the hybrid trees was predominately lodgepole pine (Fig. 5). Of the trees sampled that were designated as attacked there were eight jack pine (five from the samples from Smith, and three from Wildwood), 127 lodgepole pine, and 19 hybrids. The eight trees assigned as jack pine had *Q* values > 0.99.

## Discussion

Using simulated microsatellite genotypes and Bayesian cluster analyses, we were able to test the power of a set of microsatellites to distinguish lodgepole pine, jack pine and variable levels of hybrid ancestry. We found high agreement between the two Bayesian algorithms and low assignment error in the analysis of simulated data. Where discrepancies arose between the two methods, we generally found that Newhybrids had better power to detect hybrids while Structure performed better with pure individuals, highlighting the complementarily of the methods. While our microsatellite panel had excellent resolving power for parentals and recent hybrids, resolution declined with increasing generations of hybrid back-crossing. We expected decreased resolution based on the close evolutionary relationship between lodgepole pine and jack pine (Wheeler *et al.* 1983), however this may not be a major issue for our dataset. Given the colonization times for lodgepole pine (7000 YBP; Yeatman 1967; MacDonald & Cwynar 1985; MacDonald *et al.* 1998) and jack pine (6000 YBP; Ritchie & Yarranton 1978; McLeod & MacDonald 1997) in central Alberta, the long generation times for pine (Critchfield 1985; Muir 1993) and low hybrid vigour (Pollack 1980), the potential for geographically spread, advanced introgression is not likely (Pollack & Dancik 1985).

Our ability to distinguish species classes enabled us to address the second objective of our study, namely to determine whether jack pine have been successfully attacked by MPB. We identified eight pure jack pine trees collected from MPB attacked stands at the edge of MPB range expansion in Alberta (Figs 1 and 2). In addition, we also identified 19 hybrid trees that have also been attacked confirming their susceptibility within the hybrid zone.

The discovery that MPB has expanded its host repertoire to include jack pine has prompted us to consider whether MPB will be able to sustain eruptive populations locally and thus spread further eastward into the boreal forest. Host and beetle interaction is influenced by physiology, population dynamics and environment (Safranyik & Carroll 2006; Raffa *et al.* 2008) making this a complex system where many biotic and abiotic factors need to be considered concomitantly. This study demonstrates that both hybrids and pure jack pine are susceptible to MPB within the hybrid zone. It is currently unknown whether hybrids and jack pine have different susceptibilities to MPB attack relative to lodgepole pine. Differential susceptibility of jack pine and hybrids to MPB is plausible. For example these two species have different susceptibilities to western gall rust fungus (Yang *et al.* 1999). Also, lodgepole pine and MPB appear to share a long co-evolutionary history that has presumably allowed this tree species to adapt at some level to MPB (Raffa *et al.* 2008). In fact, Cudmore *et al.* (2010) recently showed that naïve lodgepole pine stands had higher MPB reproductive output than stands that experienced epidemic outbreaks. This suggests that MPB may have higher reproductive success in hybrid and jack pine trees which would further sustain the epidemic and support its eastward expansion. There is a potential for introgression of lodgepole pine genes – including genes that condition defence – into the jack pine genome through historical hybridization. However, widespread hybridization and introgression of defence genes seems unlikely based on our findings, therefore any potential benefits of introgression would likely be limited to the hybrid zone. As well, those trees identified as hybrids in our study exhibited higher lodgepole than jack pine ancestry (Fig. 5) suggesting a higher percentage of lodgepole pine backcrosses and limited introgression of lodgepole genes into the jack pine range. Further, chemical defences produced by lodgepole pine differ in their composition from jack pine and hybrids (Zavarin *et al.* 1969; Pollack & Dancik 1985). For example, Clark *et al.* (2008) found α-pinene, a chemical that may facilitate a successful mass attack by MPB, to be at considerably higher concentrations in jack pine than in lodgepole pine. Safranyik & Linton (1982) and Cerezke (1995) found that measures of beetle performance were similar between lodgepole pine and jack pine, suggesting the potential of jack pine to sustain populations in a manner similar to lodgepole pine. As well, there are MPB fungal associates that are part of the beetle invasion process, and it has been shown that hybrids and jack pine are susceptible (Rice *et al.* 2007a,b; Rice & Langor 2009). It should be noted that many of the published experiments were carried out using cut bolts and artificially infested rather than naturally infested live trees, limiting extrapolation to the natural boreal forest.

While tree-level defences that are partly under genetic control will conceivably contribute to the probability of MPB eruptive population dynamics in jack pine, there also needs to be a sufficient density of available hosts. The severity of the recent MPB outbreak has been partly attributed to the present-day distribution of lodgepole pine, where the continuity of even-aged stands resulting from forest management practices has been ideal for maintaining beetle populations (Taylor *et al.* 2006). In contrast, jack pine is not uniformly distributed in the boreal forest, typically occurring in a patchy distribution (Nealis & Peter 2008). Nealis & Peter (2008) and Safranyik *et al.* (2010) have analysed the potential for MPB spread in the boreal forest region and have suggested that Alberta populations are susceptible; however, jack pine occurrence may be too fragmented east of Alberta to sustain the type of epidemic that British Columbia has experienced.

Climate has influenced the severity of the recent MPB outbreak (Hicke *et al.* 2006; Régnière & Bentz 2007; Powell & Bentz 2009), and will likely play an important role in determining whether this epidemic will be maintained in the boreal forest. Temperature has received the most attention and from this there are two considerations: (i) in the past, the summer climate in northern Alberta has not been suitable to sustain synchronous beetle populations which are necessary to maintain eruptive populations (Carroll *et al.* 2003) and (ii) beetles are not completely cold tolerant and can incur high mortality within the jack pine range from cold exposure (Régnière & Bentz 2007; Cooke 2009). However, changes in global temperatures have improved the climatic suitability for both summer (Logan & Powell 2001) and winter (Carroll *et al.* 2003) seasons promoting conditions for eruptive behaviour.

The discovery of successful MPB attack and evidence of completed larval development in jack pine is a critical first step in assessing future impacts of this destructive forest pest on the boreal forest and how climate change may affect the system. We have considered whether MPB populations can be maintained in the boreal forest and there are many factors that need to be met for continued MPB expansion and population growth. If jack pine can sustain endemic populations and thus maintain this host-range expansion it is critical that forest management incorporates these considerations in their future planning. MPB is not endemic to the boreal forest and therefore should be considered an invasive species and managed as such. Forest ecosystems in North America have already been challenged with numerous pest invasions that represent a considerable threat (Liebhold *et al.* 1995). When we factor in climate change, the vulnerability of ecosystems such as the boreal forest to disturbance is further increased putting an extremely important ecosystem in jeopardy.

## Appendix I

Primer sequences and PCR conditions for 14 microsatellite loci used to type lodgepole and jack pine from British Columbia, Alberta, Saskatchewan, Ontario and Minnesota. VIC, NED, PET and 6-FAM are fluorescent dyes (Applied Biosystems)

**Table:**

Locus	Forward primer	Reverse primer	*Multiplex PCR and co-loading	Primer (μmol)	MgCl_2_ (mm)
PtTx2123†	VIC-GAAGAACCCACAAACACAAG	GGGCAAGAATTCAATGATAA	A1	0.32	1
PtTx2146 + 16bp†	VIC-TCCCCTTAAGCCTGGGGATTTGGATTGGGTATTTG	GTTTCTATATTTTCCTTGCCCCTTCCA	D2	0.96	1
PtTx3011†	6-FAM-AATTTGGGTGTATTTTTCTTAGA	AAAAGTTGAAGGAGTTGGTGATC	B1	0.64	1
PtTx3025†	NED-CACGCTGTATAATAACAATCTA	TTCTATATTCGCTTTTAGTTT	B1	0.96	1
PtTx3030†	6-FAM-AATGAAAGGCAAGTGTCG	GAGATGCAAGATAAAGGAAGTT	G2	0.64	1
PtTx3034†	NED-TCAAAATGCAAAAGACG	ATTAGGACTGGGGATGAT	C2	0.96	1
PtTx3049†	VIC-GAAGTGATAATGGCATAGCAAAAT	CAGACCCGTGAAAGTAATAAACAT	B1	0.96	1
PtTx3127†	PET-ACCCTTACTTTCAGAAGAGGATA	AATTGGGGTTCAACTATTCTATTA	A1	0.64	1
PtTx4054†	NED-TGCATTCACCTTGGAGTT	TAGGAGATAATATAAAATGTT	F3	0.64	3
PtTx4139†	6-FAM-TGGCATGCTAGGAAGAAGA	TTGTATGTTGCCTGTGGAGA	E3	0.96	1
Pcon3‡	6-FAM-CGACGAATATGTGATTGGATA	TGCTCCTAAATTTTTCAACCT	C2	0.32	1
Pcon54‡	VIC-CAGATGATGGTGTACCTTTGA	TCCAAATCTTCATTGTGTGTC	E3	0.96	1

* Loci were amplified in seven reactions (A–G) and detected in three co-loaded sets (1–3).

† Auckland et al. (2002).

‡ In house.

## Appendix II

Hardy–Weinberg calculations for sample locations where *n* ≥ 20. Included are the standard error and FIS (calculated using Weir & Cockerham 1984), all values were calculated in Genepop. *P*-values in bold are significant folowing Bonferroni correction

**Table:**

Lodgepole pine																		

	Canmore			Golden			Sparwood			Tumbler Ridge			Valemount			Willmore-Kakwa		
						
Locus	*P*-val	SE	FIS	*P*-val	SE	FIS	*P*-val	SE	FIS	*P*-val	SE	FIS	*P*-val	SE	FIS	*P*-val	SE	FIS
PtTx2123	0.354	0.004	−0.068	0.376	0.004	0.113	0.637	0.003	0.111	0.225	0.004	−0.076	0.360	0.003	0.172	0.101	0.002	−0.406
PtTx3030	0.735	0.005	0.040	**0.000**	0.000	0.470	**0.000**	0.000	0.493	**0.000**	0.000	0.549	**0.000**	0.000	0.553	0.094	0.003	0.165
PtTx3127	0.019	0.002	0.275	0.253	0.005	0.020	0.859	0.002	−0.015	0.468	0.006	0.111	0.098	0.003	0.169	0.451	0.004	0.088
PtTx3011	**0.000**	0.000	0.223	0.034	0.005	0.150	0.075	0.008	0.135	0.138	0.010	0.127	0.060	0.006	0.157	**0.001**	0.000	0.246
PtTx3049	**0.002**	0.001	0.198	0.023	0.003	0.134	0.401	0.010	0.124	**0.000**	0.000	0.359	0.018	0.002	0.167	**0.000**	0.000	0.463
PtTx3025	0.776	0.007	−0.089	0.990	0.001	−0.171	0.534	0.008	−0.086	0.151	0.005	0.066	0.408	0.010	0.029	0.401	0.008	−0.171
Pcon3	0.026	0.003	0.300	0.037	0.005	0.167	0.034	0.004	0.102	0.033	0.004	0.104	0.045	0.004	0.097	0.003	0.001	0.238
PtTx3034	0.875	0.004	0.077	0.010	0.001	0.212	**0.000**	0.000	0.393	0.880	0.004	0.029	0.061	0.003	0.276	**0.001**	0.000	0.482
PtTx2146	0.491	0.011	0.034	0.442	0.010	−0.050	0.502	0.010	−0.080	0.041	0.004	0.139	0.483	0.009	0.024	0.661	0.010	−0.004
PtTx4139	0.784	0.008	0.047	**0.000**	0.000	0.296	0.036	0.004	0.224	0.344	0.009	0.095	0.293	0.009	0.131	**0.000**	0.000	0.227
PtTx4054	0.871	0.006	0.029	0.528	0.009	0.000	0.085	0.006	0.018	0.321	0.009	0.078	0.007	0.002	0.188	0.118	0.007	0.133

Jack pine																		

	Conklin			FtMcMurray			Ontario/Minnesota			Saskatchewan								
										
Locus	P-val	SE	FIS	P-val	SE	FIS	P-val	SE	FIS	P-val	SE	FIS						
PtTx2123	0.767	0.003	0.052	0.858	0.002	−0.039	0.333	0.002	0.068	0.612	0.003	0.099						
PtTx3030	**0.002**	0.001	0.286	**0.000**	0.000	0.337	**0.001**	0.001	0.320	0.056	0.004	0.265						
PtTx3127	1.000	0.000	−0.015	**0.001**	0.000	0.432	1.000	0.000	−0.031	1.000	0.000	−0.017						
PtTx3011	**0.002**	0.001	0.153	0.014	0.003	0.126	0.329	0.010	0.075	0.020	0.003	0.171						
PtTx3049	0.074	0.003	0.083	0.014	0.002	0.232	0.107	0.003	0.147	0.732	0.004	0.003						
PtTx3025	0.288	0.010	0.074	0.207	0.007	−0.062	0.036	0.001	0.190	0.406	0.005	0.156						
Pcon3	0.877	0.005	0.004	0.235	0.009	0.124	0.916	0.004	0.016	0.358	0.008	0.115						
PtTx3034	**0.000**	0.000	0.412	**0.000**	0.000	0.465	**0.002**	0.000	0.286	0.000	0.000	0.455						
PtTx2146	0.024	0.003	−0.050	0.261	0.008	−0.068	0.578	0.006	0.024	0.131	0.005	−0.071						
PtTx4139	0.616	0.012	0.028	0.170	0.009	−0.017	0.844	0.006	−0.050	0.006	0.002	0.106						
PtTx4054	0.459	0.012	−0.014	0.727	0.010	−0.084	0.029	0.002	0.162	1.000	0.000	−0.099						

## Appendix III

Allelic diversity measures for all data and for only samples that were assigned to jack pine and lodgepole pine. Number of alleles (N), allelic richness (Na), effective number of alleles (Ne), observed heterozygosity (H_O_), expected heterozygosity (H_E_) and the fixation index (F) were calculated in GenAlEx 6 (Peakall & Smouse 2006). Allelic richness (Richness) and private allelic richness (Private) were calculated using rarefaction in hp-rare 1.0 (Kalinowski 2005)

**Table:**

Pop	FIS	Locus	N	Na	Ne	Ho	He	F	Richness	Private
All	0.242	PtTx2123	677	6	4.038	0.600	0.752	0.203	6.0	
		PtTx3030	667	22	5.160	0.465	0.806	0.424	21.9	
		PtTx3127	662	12	2.397	0.384	0.583	0.342	11.9	
		PtTx3011	673	49	26.051	0.798	0.962	0.170	48.8	
		PtTx3049	649	19	8.980	0.695	0.889	0.218	18.9	
		PtTx3025	674	22	5.061	0.706	0.802	0.120	21.8	
		Pcon3	651	32	11.475	0.768	0.913	0.159	31.9	
		PtTx3034	649	17	5.506	0.522	0.818	0.362	17.0	
		PtTx2146	668	24	8.130	0.823	0.877	0.061	24.6	
		PtTx4139	673	25	8.250	0.722	0.879	0.178	24.6	
		Pcon54	673	17	4.004	0.429	0.750	0.428	16.9	
		PtTx4054	675	18	5.055	0.612	0.802	0.237	18.0	
Jack	0.172	PtTx2123	299	4	2.331	0.548	0.571	0.039	4.0	0.0
		PtTx3030	294	16	1.885	0.327	0.469	0.304	15.4	6.1
		PtTx3127	287	5	1.096	0.070	0.088	0.207	4.9	0.0
		PtTx3011	299	31	12.353	0.793	0.919	0.138	30.2	8.1
		PtTx3049	281	12	5.135	0.683	0.805	0.151	11.9	0.0
		PtTx3025	299	14	2.919	0.599	0.657	0.089	13.5	1.1
		Pcon3	295	16	5.939	0.786	0.832	0.054	15.4	0.0
		PtTx3034	285	12	2.952	0.400	0.661	0.395	11.7	1.0
		PtTx2146	299	14	4.330	0.796	0.769	−0.035	13.8	1.0
		PtTx4139	298	17	3.468	0.688	0.712	0.033	16.8	0.8
		Pcon54	298	11	2.308	0.168	0.567	0.704	10.5	1.0
		PtTx4054	299	14	1.451	0.314	0.311	−0.012	13.7	0.1
Lodgepole	0.122	PtTx2123	279	6	2.553	0.624	0.608	−0.025	6.0	2.0
		PtTx3030	274	15	4.732	0.544	0.789	0.310	14.6	5.4
		PtTx3127	277	12	3.196	0.635	0.687	0.075	11.8	6.9
		PtTx3011	276	41	19.515	0.804	0.949	0.152	40.5	18.4
		PtTx3049	275	19	10.611	0.695	0.906	0.233	18.8	6.9
		PtTx3025	277	21	4.081	0.791	0.755	−0.047	20.7	8.3
		Pcon3	261	30	13.396	0.747	0.925	0.193	29.9	14.5
		PtTx3034	268	15	5.730	0.631	0.825	0.236	14.9	4.1
		PtTx2146	272	23	6.257	0.824	0.840	0.020	22.4	9.7
		PtTx4139	277	22	11.065	0.755	0.910	0.171	21.8	5.9
		Pcon54	277	16	3.884	0.675	0.743	0.091	15.9	6.4
		PtTx4054	278	18	9.759	0.849	0.898	0.054	17.9	4.3
